# Synthesis of Novel *N*^4^-Hydrocytidine Analogs as Potential Anti-SARS-CoV-2 Agents

**DOI:** 10.3390/ph15091144

**Published:** 2022-09-14

**Authors:** Franck Amblard, Julia C. LeCher, Ramyani De, Shu Ling Goh, Chengwei Li, Mahesh Kasthuri, Nicolas Biteau, Longhu Zhou, Zahira Tber, Jessica Downs-Bowen, Keivan Zandi, Raymond F. Schinazi

**Affiliations:** Center for ViroScience/Cure, Laboratory of Biochemical Pharmacology, Department of Pediatrics, Emory University School of Medicine, and Children’s Healthcare of Atlanta, 1760 Haygood Drive, Atlanta, GA 30322, USA

**Keywords:** nucleoside, antiviral agents, COVID-19

## Abstract

Coronavirus disease 2019 (COVID-19) is an emerging global pandemic with severe morbidity and mortality caused by the severe acute respiratory syndrome coronavirus 2 (SARS-CoV-2). Molnupiravir, an ester prodrug form of *N*^4^-hydroxycytidine (NHC), was recently emergency-use approved for the treatment of early SARS-CoV-2 infections. Herein, we report the synthesis and evaluation of a series of novel NHC analogs.

## 1. Introduction

The coronavirus pandemic has caused a dual threat to the health and the economy of the U.S. and the world. COVID-19 was first identified in late 2019 in Wuhan, Hubei province, China, resulting in the ongoing 2019–2022 pandemic. COVID-19 is caused by the severe acute respiratory syndrome coronavirus 2 (SARS-CoV-2). Common symptoms of the disease include fever (88%), dry cough (68%), shortness of breath (19%), and loss of smell (15 to 30%) as well as complications such as pneumonia, bronchitis, viral sepsis, diarrhea, and acute respiratory distress syndrome [1,2]. SARS-CoV-2 is the seventh human coronavirus after 229E, NL63, OC43, HKU1, MERS-CoV, and the original SARS-CoV. Like all coronaviruses, SARS-CoV-2 is an enveloped, positive-sense, single-stranded RNA virus of approximately 30,000 bases in length. Based on the genome organization of SARS-CoV-2, four enzymes are recognized as attractive drug targets, which include the 3CLpro (nsp5), the PLpro (nsp3), RNA helicase (nsp13), and the RNA-dependent RNA polymerase (RdRp, nsp12). The RdRp catalyzes the synthesis of viral RNA and thus plays a central role in the replication and transcription cycle of CoV-2 [3].

**Figure 1:**
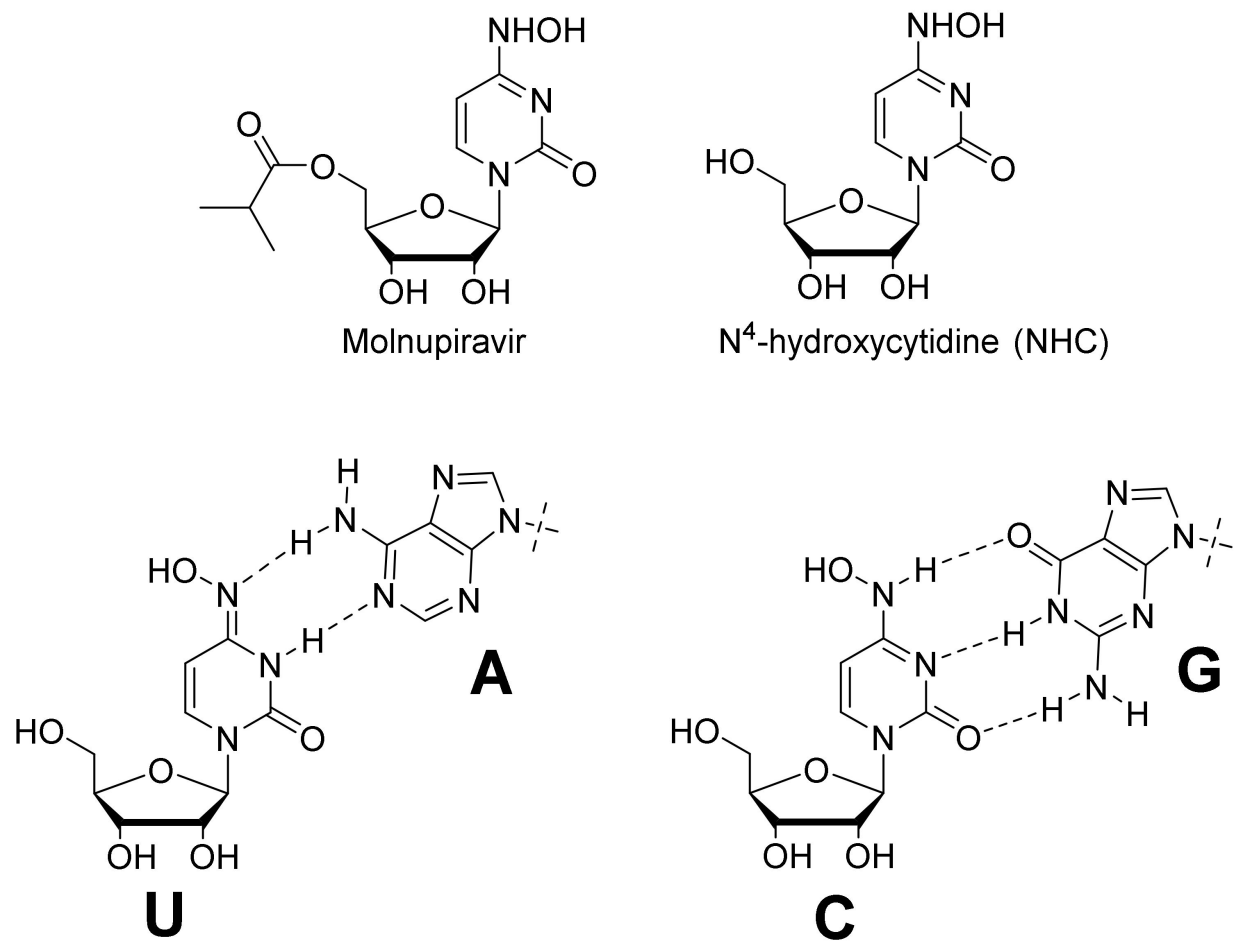
Structure of molnupiravir, the isobutyryl ester prodrug of N4-hydroxycytidine (NHC) and the U-A or C-G base pairing of NHC’s tautomeric forms.

Remdesivir was the first nucleoside analog RdRp inhibitor approved by the FDA for the treatment of SARS-CoV-2-infected patients, but its modest to no effect on hospitalization and mortality [4,5] as well as its poor pharmacokinetic properties (it is currently approved for intravenous administration and is therefore limited to hospitalized patients), make remdesivir monotherapy a sub-par option to treat SARS-CoV-2 -infected individuals. Molnupiravir (EIDD-2801), the 5′-isobutyryl ester prodrug form of *N*^4^-hydroxycytidine (NHC), was emergency-use authorized by the FDA for the treatment of COVID-19 in certain adults, but despite its potent in vitro activity, it shows significant toxicity in in certain cell-based systems, including the induction of mutagenesis in mammalian cells [6,7,8,9]. Consequently, molnupiravir’s approval came with a multitude of limitations, including being restricted to patients older than 18 years of age due to its effect on bone and cartilage growth [10] and to non-pregnant women due to potential fetal harm [11]. Finally, it is worth noting that when treatment with molnupiravir was conducted in a well-controlled study, it decreased the risk of hospitalization from COVID-19 by only 30% and its benefit has not been observed in subjects when treatment was initiated after hospitalization due to COVID-19 [12]. The key feature of molnupiravir/NHC is its 4-oxime group, which can either mimic a uridine base pairing with adenosine or a cytidine base pairing with guanosine, depending on its tautomer form (Figure 1).

The incorporation of NHC 5′-triphosphate (the active form of molnupiravir) by the viral (and cellular) RdRp during transcription of the viral genome leads to RNA mutations and impairs SARS-CoV-2 replication. Based on this unique attribute, and in order to mitigate molnupiravir’s above mentioned liabilities and limitations, we prepared a series of 4-NHOH pyrimidine nucleoside analogs (Compounds **1**–**13**, see Table 1 for structures) and herein report on their evaluation against SARS-CoV-2 in culture.

## 2. Results and Discussion

### 2.1. Chemistry

**Scheme 1:**
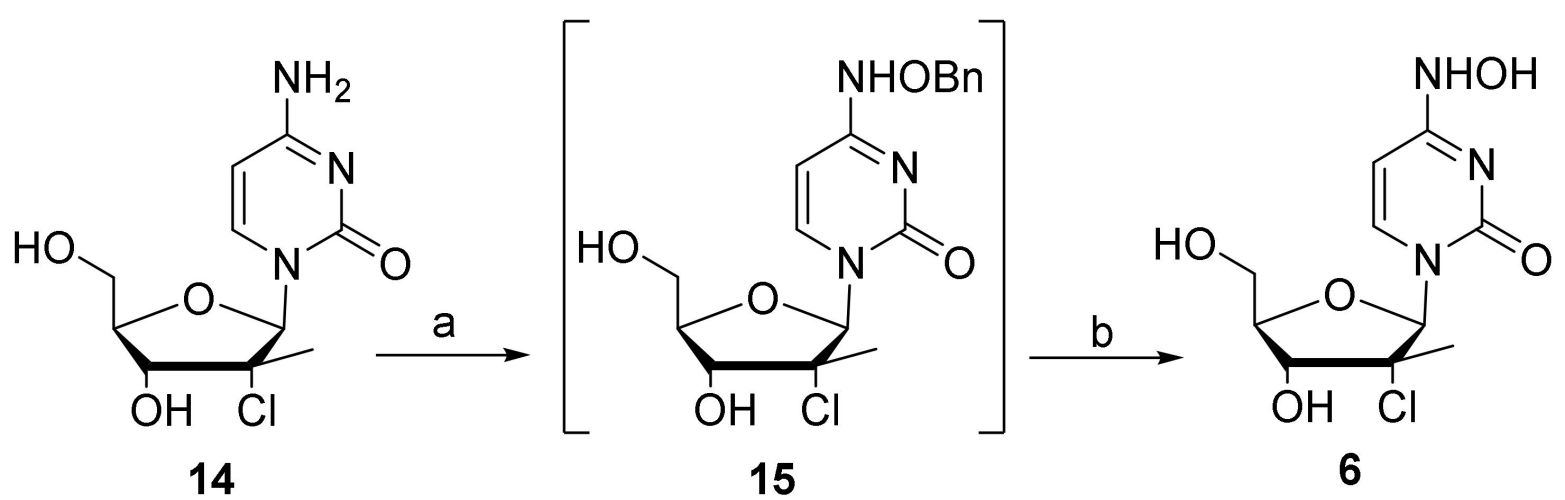
(**a**) BnONH_2_.HCl, dioxane/water, reflux, overnight; (**b**) cyclohexadiene, Pd/C, room temperature, overnight, 10%.

*N^4^*-Hydroxy-2′-deoxycytidine **1** [13], *N^4^*-hydroxy-2′-deoxy-2′-difluorocytidine **2** [14], *N^4^*-hydroxy-2′-deoxy-2′-fluorocytidine **3** [14], *N^4^*-hydroxy-2′-C-methylcytidine **4** [15], *N^4^*-hydroxy-2′-deoxy-2′-fluoro-2′-C-methylcytidine **5** [15], *N^4^*-hydroxy-4′-fluorocytidine **7** [16], *N^4^*-hydroxy-6-azacytidine **10** [17], *N^4^*-hydroxycytosine dioxolane **11** [18], and L- *N^4^*-hydroxycytidine **13** [14] were prepared according to reported procedures. On the other hand, *N^4^*-hydroxy-2′-deoxy-2′-fluoro-2′-C-chlorocytidine **6** was prepared by reaction of 2′-deoxy-2′-fluoro-2′-C-chlorocytidine **14** [19] with benzyloxy amine in dioxane and water and underwent further deprotection using cyclohexadiene as a source of hydrogen in the presence of Pd/C (Scheme 1).

**Scheme 2:**
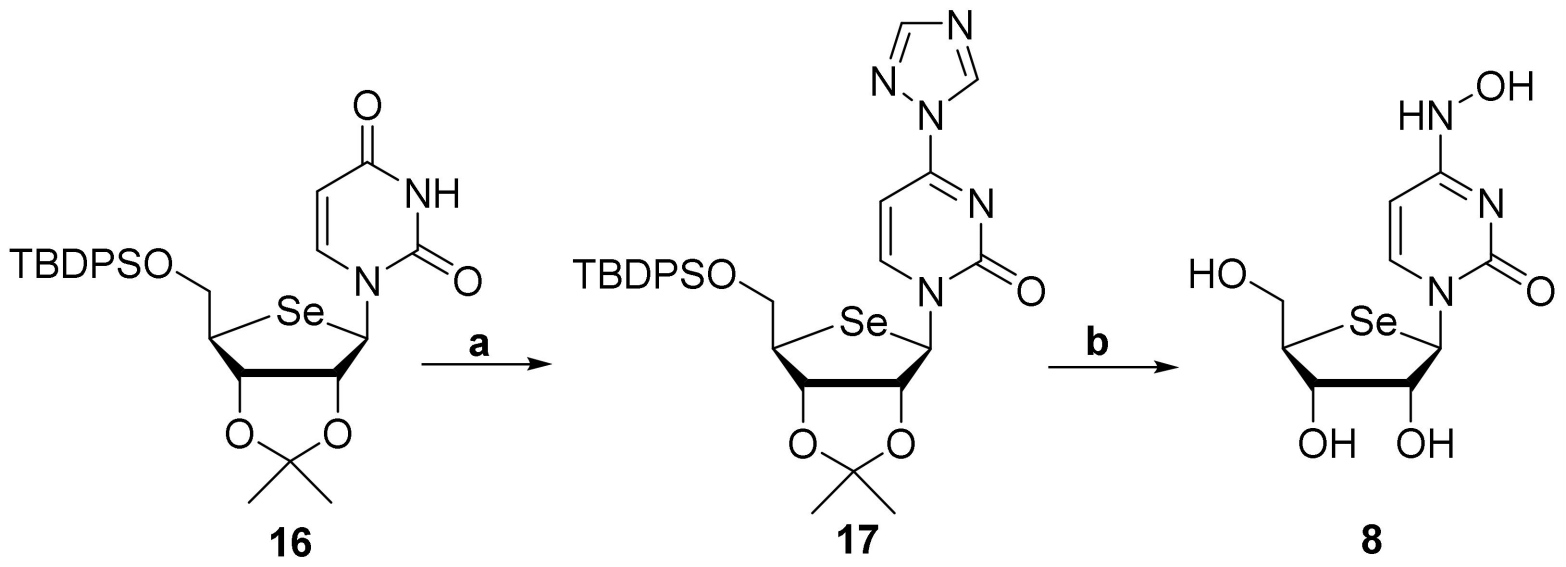
(**a**) POCl_3_, Et_3_N, 1,2,4-triazole, 0 °C to rt, 12 h, 68%. (**b**) NH_2_OH 50% in H_2_O, ACN, rt, 20 min; then 50% aq TFA, rt, 2 h, 49% over 2 steps.

*N*^4^-Hydroxy-selenoriboside cytidine **8** was prepared according to the chemistry described in Scheme 2. Compound **16** [20] was reacted with POCl_3_ and 1,2,4-triazole in the presence of Et_3_N to give 4-triazolo intermediate **17** which was reacted with hydroxylamine for 20 min and then deprotected under acidic conditions to obtain the desired compound **8** in 49% yield over two steps.

**Scheme 3:**
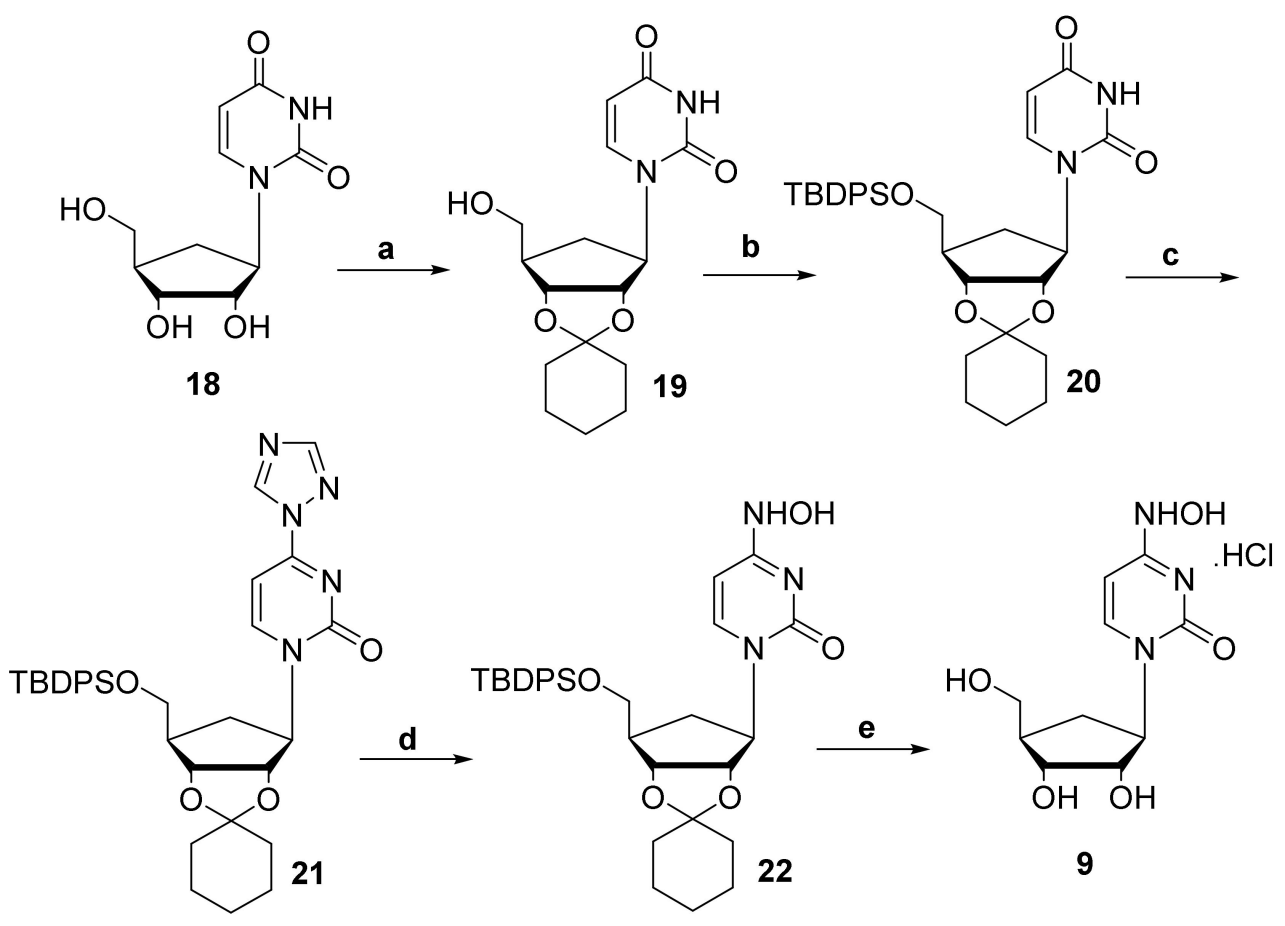
(**a**) *p*-TSA, cyclohexanone, 4 A MS, acetone, rt, 5 h, 82%. (**b**) TBDPSCl, imidazole, THF, 0 °C to rt, 24 h, 85%. (**c**) POCl_3_, Et_3_N, 1,2,4-triazole, 0 °C to rt, overnight, 60%. (**d**) NH_2_OH 50% in H_2_O, H_2_O/ACN, rt, 20 min, 71%. (**e**) 1M HCl, MeOH, 0 °C to rt, 5 h, 82%.

Compound **9**, the carbocyclic version of NHC, was prepared by following the chemistry described in Scheme 3. Carbocyclic uracil analog **18** [21] was 2′,3′-protected using cyclohexanone in the presence of *p*-toluenesulfonic acid (p-TSA) and then 5′-protected using *tert*-butyl(chloro)diphenylsilane (TBDPSCl) in the presence of 4-dimethylaminopyridine (DMAP). Compound **20** was then reacted with POCl_3_ and 1,2,4-triazole in the presence of triethylamine to form 4-triazolo intermediate **21** which was reacted with hydroxylamine and then deprotected under acidic condition to obtain the desired compound **9**.

**Scheme 4:**
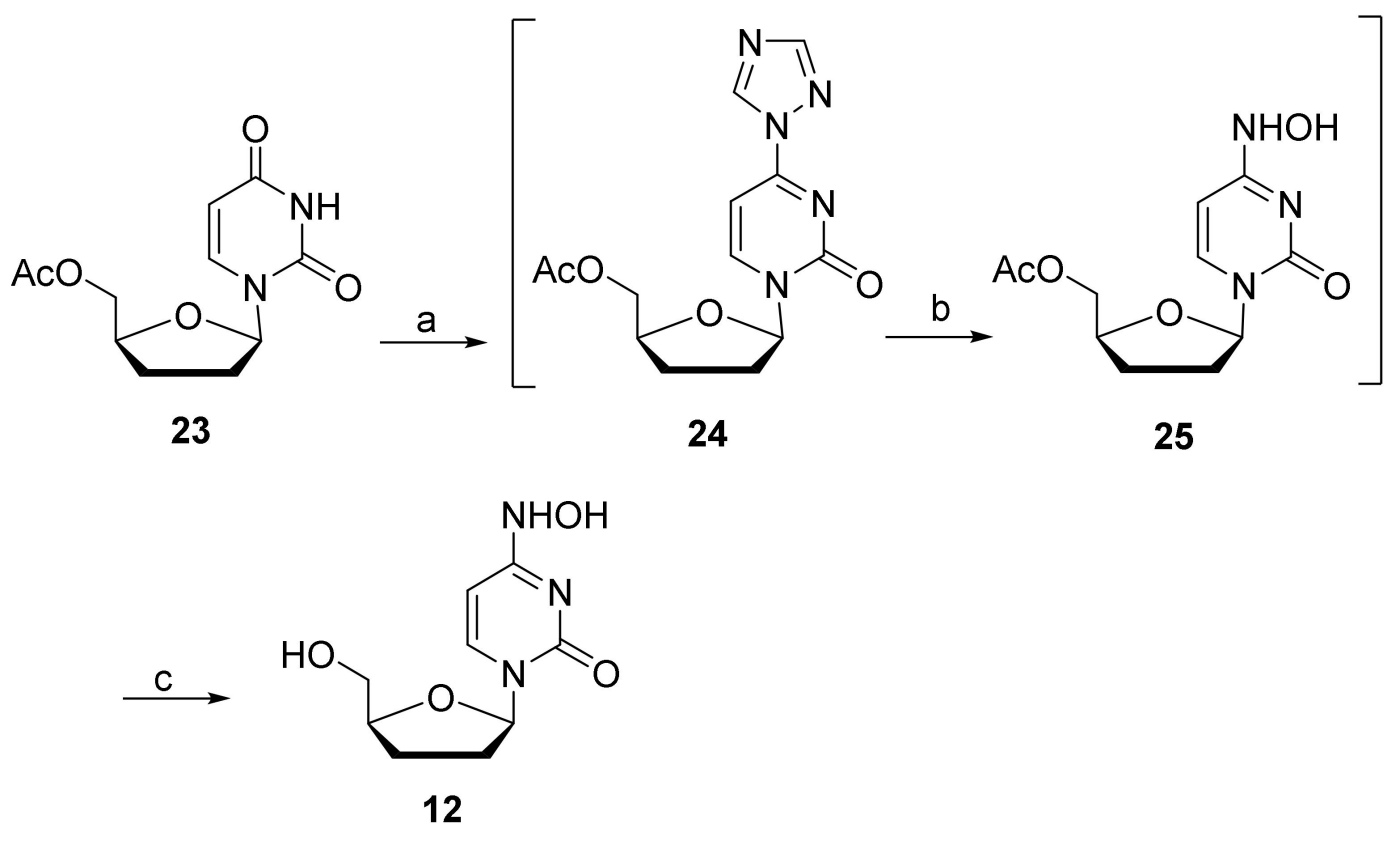
(**a**) 4-Chlorophenyl dichlorophosphate, 1,2,4-triazole, pyridine, rt, 4 days. (**b**) NH_2_OH 50% in H_2_O, ACN, rt, 2 h. (**c**) NH_3_/MeOH, rt, overnight, 15% over 3 steps.

*N*^4^-Hydroxy-2′,3′-dideoxycytidine **12** was prepared according to the chemistry described in Scheme 4. 2′,3′-Dideoxy,5′-OAc-cytidine **23** [22] was first reacted with 4-chlorophenyl dichlorophosphate and 1,2,4-triazole in the presence of pyridine and then treated with hydroxylamine to give the intermediate **25**. Final deprotection of crude compound **25** in a saturated solution of methanolic ammonia gave the targeted compound **12**.

### 2.2. Antiviral Evaluation

The anti-SARS-CoV-2 activity of the 4-NHOH nucleoside analogs **1**–**13** herein prepared was evaluated at 10 µM following previously reported methods [23]. Briefly, a monolayer of Vero cells in a 96-well cell culture microplate was treated with 10 µM of each compound for 1 h followed by infection with SARS-CoV-2 at 0.1 MOI [24]. After 1 h adsorption at 37 °C, the virus inoculum was removed and the compound or vehicle-containing medium was added to the respected wells. Resultant virus progeny yield was measured 2 days post-treatment from the supernatant of treated infected cells by specific quantitative RT-PCR. While NHC displayed more than 99% inhibition at 10 µM in our assay, compounds **1**–**13** exhibited no significant inhibition at that same concentration (Table 1). Interestingly, even a small modification of NHC’s base (6-aza derivative 10) or a minor modification of its sugar ring (seleno or carbocyclic sugar derivatives **8** and **9**, 2′-modified compounds 2–6 or 4′-fluorinated analog **7**) completely abrogated NHC’s anti-SARS-CoV-2 activity. Similarly, L-NHC (13), 2′,3′-dideoxy compound **12** and dioxolane analog **11** were inactive at the maximum concentration tested. It is worth noting that none of these compounds displayed toxicity in Vero cells at concentration up to 100 μM while NHC had a CC_50_ of 16 μM in these cells.

## 3. Materials and Methods

### 3.1. General Information

Anhydrous solvents were purchased from Millipore Sigma (Milwaukee, WI, USA). All commercially available reagents were used without further purification. Reagents were purchased from commercial sources. All the reactions were carried out under nitrogen in oven-dried glassware unless otherwise noted. Thin layer chromatography was performed on Analtech GHLF silica gel plates. Column chromatography was accomplished on Combiflash Rf200 or via reverse-phase high-performance liquid chromatography. ^1^H, ^13^C, and ^19^F NMR spectra were recorded on a Bruker Ascend 400 spectrometer at rt (400, 101, and 377 MHz) and residual proton solvent signals were used as internal standards. Deuterium exchange and decoupling experiments were utilized to confirm proton assignments. NMR processing was performed with MestReNova (Mestrelab Research, Compostela, Spain) version 14.1.1 24571 or Topspin (Bruker, Berlin, Germany) version 3.5. Signal multiplicities are represented by s (singlet), d (doublet), dd (doublet of doublets), t (triplet), q (quadruplet), br (broad), bs (broad singlet), and m (multiplet). Coupling constants (*J*) are in hertz (Hz). Mass spectra were determined on a Waters Acquity ultraperformance liquid chromatography (UPLC) spectrometer using a SQ detector with electrospray ionization. The purity of final compounds was determined to be >95% using UPLC analyses performed on a Waters Acquity UPLC System with a Kinetex LC column (2.1 mm, 50 mm, 1.7 μm, C18, 100 Å) and further supported by clean NMR spectra. Mobile phase flow was 0.4 mL/min with a 1.20 min gradient from 95% aqueous media (0.05% formic acid) to 95% CH_3_CN (0.05% formic acid) and a 4.5 min total acquisition time. Photodiode array detection was from 190 to 360 nm.

### 3.2. Chemistry

***N*^4^-Hydroxy-2′-deoxy-2′-fluoro-2′-C-chlorocytidine (6):**

A solution of 2′-deoxy-2′-fluoro-2′-C-chlorocytidine **14** (200 mg, 0.73 mmol) and *N*^4^-benzylhydroxylamine hydrochloride (750 mg, 4.7 mmol) in dioxane (5 mL) and water (5 mL) was heated at 100 °C overnight. Volatiles were removed under vacuum and the residue was purified by flash chromatography (DCM to DCM/MeOH, 20/1) to obtain *N*^4^-benzylhydroxylamine intermediate **15**. This intermediate (75 mg, 0.195 mmol) in EtOH (19 mL) was hydrogenated using Pd/C (75 mg) and cyclohexadiene (0.75 mL) overnight. The volatiles were removed under vacuum and the residue purified by flash chromatography (CH_2_Cl_2_/MeOH, 100/0 to 90/10) to give compound **6** (19 mg, 10% over 2 steps). ^1^H NMR (CD_3_OD, 400 MHz) *δ* 8.05 (m, 4H), 7.49 (d, 1H, *J* = 8.3 Hz), 6.34 (s, 1H), 5.60 (d, 1H, *J* = 8.3 Hz), 3.90–4.04 (m, 3H), 3.80 (dd, 1H, *J* = 12.5, 2.2 Hz), 1.56 (s, 3H). ^13^C NMR (CD_3_OD, 400 MHz) *δ* 150.2, 144.6, 129.9, 97.7, 91.6, 82.2, 77.4, 72.6, 58.5, 21.6. HRMS for C_10_ClH_15_N_3_O_5_ (M+H): *m*/*z*: cacld: 292.0700. found: *m*/*z*: 292.0701.

**1-((3a*R*,4*R*,6*R*,6a*S*)-6-(((*tert*-Butyldiphenylsilyl)oxy)methyl)-2,2-dimethyltetrahydroselenopheno [3,4-*d*][1,3]dioxol-4-yl)-4-(1*H*-1,2,4-triazol-1-yl)pyrimidin-2(1*H*)-one (17):**

To a suspension of 1,2,4-triazole (1.34 g, 19.5 mmol) in acetonitrile (30 mL) at 0 °C was added POCl_3_ (300 μL, 3.2 mmol) followed by triethylamine (2.92 mL, 20.9 mmol) and the resulting reaction mixture was stirred at 0 °C for 1 h. A solution of **16** (300 mg, 0.57 mmol) in acetonitrile (6 mL) was then added to the previous solution and the reaction mixture was slowly warmed to room temperature and stirred for 12 h. The reaction was quenched with saturated aq. NaHCO_3_ (5 mL) and extracted with dichloromethane (30 mL × 3). The combined organic layers were washed with brine (10 mL), dried over NaSO_4_, filtered, and concentrated under reduced pressure. The crude product was purified by flash column chromatography (0–40% ethyl acetate/hexane, 0/100 to 40/60) to give compound **17** (314 mg, 86%). ^1^H NMR (400 MHz, CDCl_3_): *δ* 9.24 (s, 1H), 8.25 (d, *J* = 7.2 Hz, 1H), 8.12 (s, 1H), 7.67 (m, 4H), 7.40 (m, 6H), 6.76 (d, *J* = 7.2Hz, 1H), 6.42 (d, *J* = 3.2 Hz, 1H), 4.87 (t, *J* = 2.8 Hz, 1H), 4.81 (t, *J* = 3.2 Hz, 1H), 4.06 (m, 3H), 1.56 (s, 3H), 1.30 (s, 3H), 1.09 (s, 9H). ^13^C NMR (101 MHz, CDCl_3_): *δ* 158.9, 154.2, 154.0, 149.0, 143.3, 135.6, 135.5, 133.0, 132.6, 130.1, 127.9, 112.0, 95.4, 90.9, 86.0, 65.9, 63.3, 52.0, 27.7, 26.9, 25.2, 19.3. HRMS (ESI): *m*/*z* [M+H] ^+^ calcd. for C_30_H_35_N_5_O_4_SeSi: 638.1624, found: 638.170.

**1-((2*R*,3*R*,4*S*,5*R*)-3,4-Dihydroxy-5-(hydroxymethyl)tetrahydroselenophen-2-yl)-4-(hydroxyamino)pyrimidin-2(1*H*)-one (8):**

To a solution of **17** (100 mg, 0.157 mmol) in acetonitrile (1 mL) was added hydroxylamine (50% in water, 627 μL, 0.314 mmol) at room temperature. The resulting reaction mixture was stirred for 20 min before evaporation of the volatiles. The crude product was then treated with 50% aq. TFA (2.5 mL) at room temperature for 2 h. The volatiles were then removed under vacuum and the residue purified by flash column chromatography (methanol/dichloromethane, 0/100 to 10/90) to obtain compound **8** (32.4 mg, 64%). ^1^H NMR (400 MHz, DMSO-d_6_): *δ* 9.97 (s, 1H), 9.52 (d, *J* = 0.8 Hz, 1H), 7.13 (d, *J* = 8 Hz, 1H), 6.07 (d, *J* = 8.8 Hz, 1H), 5.65 (d, *J* = 8 Hz, 1H), 5.29 (bro s, 1H), 5.22 (bro s, 1H), 5.14 (bro s, 1H), 4.17 (d, *J* = 8.4 Hz, 1H), 4.11 (s, 1H), 3.71 (t, *J* = 5.2 Hz, 1H), 3.55 (t, *J* = 5.2 Hz, 1H), 33.3 (t, *J* = 1.6 Hz, 1H). ^13^C NMR (101 MHz, CDCl_3_): *δ* 149.1, 142.9, 130.4, 98.2, 75.9, 73, 63.5, 54.9, 48. HRMS (ESI): *m*/*z* [M+H] ^+^ calcd. for C_9_H_13_N_3_O_5_Se: 324.0020, found: 324.0097.

**1-((3*a*′*R*,4′*R*,6′*R*,6*a*′*S*)-4′-(Hydroxymethyl)tetrahydro-4′*H*-spiro[cyclohexane-1,2′-cyclopenta[*d*][1,3]dioxol]-6′-yl)pyrimidine-2,4(1*H*,3*H*)-dione (19):**

To a solution of **18** (100 mg, 0.413 mmol) in cyclohexanone (0.7 mL), *p*-TSA was added (7.2 mg, 0.041 mmol) at room temperature. The resulting reaction mixture was stirred overnight before addition of triethylamine (0.072 mL, 0.516 mmol). After evaporation of the volatiles under vacuum, the residue was purified by flash column chromatography (ethyl acetate/hexane, 0/100 to 100/0) to obtain compound **19** (109 mg, 82%). ^1^H NMR (400 MHz, Acetone-*d_6_*) *δ* 10.02 (bs, 1H), 7.67 (d, 1H, *J* = 8.0 Hz), 5.60 (d, 1H, *J* = 8.0 Hz), 4.83–4.75 (m, 2H), 4.53 (dd, 1H, *J* = 4.8, 1.6 Hz), 3.90–3.80 (m, 1H), 3.67–3.63 (m, 2H), 2.26–2.23 (m, 2H), 2.60–1.97 (m, 1H), 1.73–1.70 (m, 2H), 1.64–1.59 (m, 2H), 1.52–1.49 (m, 4H), 1.39–1.35 (m, 2H). ^13^C NMR (101 MHz, Acetone-*d_6_*) *δ* 164.4, 152.6, 144.6, 114.9, 103.4, 84.1, 82.5, 64.8, 64.4, 47.7, 39.3, 36.4, 34.2, 26.7, 25.6, 25.1. HRMS-ESI (*m*/*z*) [M+H]^+^ calcd. for C_16_H_23_N_2_O_5_: 324.1529, found: 324.1553.

**1-((3*a*′*R*,4′*R*,6′*R*,6*a*′*S*)-4′-(((tert-Butyldiphenylsilyl)oxy)methyl)tetrahydro-4′*H*-spiro[cyclohexane-1,2′-cyclopenta[*d*][1,3]dioxol]-6′-yl)pyrimidine-2,4(1*H*,3*H*)-dione (20):**

To a solution of **19** (100 mg, 0.311 mmol) in *N,N*-dimethylformamide (3 mL), imidazole (102 mg, 1.55 mmol) was added at room temperature. The resulting reaction mixture was stirred for 15 min at 0 °C before dropwise addition of TBDPSCl (0.21 mL, 0.776 mmol). The mixture was kept at 0 °C for 10 more minutes and then stirred 24 h at room temperature. After removal of the volatiles under vacuum, the residue was diluted with EtOAc (20 mL), washed with a saturated solution of NaHCO_3_ (20 mL), water (10 mL), and brine (10 mL). The organic phase was concentrated under vacuum and the residue purified by flash column chromatography (ethyl acetate/hexane, 0/100 to 100/0) to obtain compound **20** (152 mg, 85%). ^1^H NMR (400 MHz, Acetone-*d_6_*) *δ* 10.02 (bs, 1H), 7.74–7.71 (m, 4H), 7.62 (d, 1H, *J* = 8.0 Hz), 7.48–7.42 (m, 6H), 5.58 (d, 1H, *J* = 8.0 Hz), 4.86 (dd, 1H, *J* = 5.4, 1.5 Hz), 4.76–4.70 (m, 1H), 4.59 (t, 1H, *J* = 7.2 Hz), 3.84 (d, 2H, *J* = 6 Hz), 2.39–2.18 (m, 2H), 1. 97–1.94 (m, 1H), 1.79–1.72 (m, 2H), 1.66–1.58 (m, 1H), 1.57–1.51 (m, 4H), 1.42–1.31 (m, 2H), 1.07 (bs, 9H). ^13^C NMR (101 MHz, Acetone-*d_6_*) *δ* 164.4, 152.6, 144.9, 137.3, 135.3, 131.5, 129.5, 115.1, 103.4, 84.0, 82.5, 66.6, 65.1, 48.0, 39.2, 36.4, 34.2, 29.9, 28.1, 26.7, 25.6, 25.1, 20.8. HRMS-ESI (*m*/*z*) [M+H]^+^ calcd. for C_32_H_41_N_2_O_5_Si: 561.2706, found: 561.2784.

**1-((3*a′R*,4′*R*,6′*R*,6a*′S*)-4′-(((tert-Butyldiphenylsilyl)oxy)methyl)tetrahydro-4′H-spiro[cyclohexane-1,2′-cyclopenta[*d*][1,3]dioxol]-6′-yl)-4-(1*H*-1,2,4-triazol-1-yl)pyrimidin-2(1*H*)-one (21):**

To a solution of 1,2,4-triazole (371 mg, 5.38 mmol) in acetonitrile (10 mL), Et_3_N (100 mL, 0.157 mmol) and phosphoryl chloride (0.084 mL, 0.903 mmol) were added at 0 °C. The mixture was stirred at 0 °C for 3 h before dropwise addition of a solution of **20** (100 mg, 0.174 mmol) in acetonitrile (2 mL) at 0 °C. The reaction mixture was stirred overnight at room temperature and then diluted with EtOAc (30 mL). The resulting mixture was filtered off and washed with a saturated solution of NaHCO_3_ (15 mL) and brine (10 mL). The organic phase was concentrated under vacuum and the residue purified by flash column chromatography (ethyl acetate/hexane, 0/100 to 80/20) to obtain compound **21** (32.4 mg, 60%).^1^H NMR (400 MHz, Acetone-*d_6_*) *δ* 9.24 (s, 1H), 8.45 (d, 1H, *J* = 8.0 Hz), 8.23 (s, 1H), 7.75–7.73 (m, 4H), 7.48–7.42 (m, 6H), 6.99 (d, 1H, *J* = 8.0 Hz), 5.02 (dd, 1H, *J* = 5.0, 1.9 Hz), 4.88–4.83 (m, 1H), 4.68 (dd, 1H, *J* = 5.4, 1.3 Hz), 3.88–3.81 (m, 2H), 2.43–2.2 (m, 3H), 1.74–1.71 (m, 2H), 1.65–1.50 (m, 6H), 1.39–1.34 (m, 2H)., 1.09 (bs, 9H). ^13^C NMR (101 MHz, Acetone-*d_6_*) *δ* 160.8, 155.9, 155.2, 153.2, 144.9, 137.3, 135.3, 131.5, 129.5, 115.1, 103.4, 95.6, 84.0, 82.8, 68.9, 66.1, 48.5, 39.3, 36.4, 34.2, 28.1, 26.7, 25.6, 25.1, 20.8. HRMS-ESI (*m*/*z*) [M+H]^+^ calcd. for C_34_H_42_N_5_O_4_Si: 612.2928, found 612.3007.

**1-((3*a′R*,4′*R*,6′*R*,6*a′S*)-4′-(((tert-Butyldiphenylsilyl)oxy)methyl)tetrahydro-4′*H*-spiro[cyclohexane-1,2′-cyclopenta[*d*][1,3]dioxol]-6′-yl)-4-(hydroxyamino)pyrimidin-2(1*H*)-one (22):**

To a solution of **21** (100 mg, 0.154 mmol) in acetonitrile (0.62 mL), hydroxylamine 50% in water (0.019 mL, 0.308 mmol) was added at room temperature. The resulting reaction mixture was stirred for 20 min before evaporation of the volatiles. The residue was then purified by flash column chromatography (methanol/dichloromethane, 0/100 to 10/90) to give compound **22** (66.8 mg, 71%). ^1^H NMR (400 MHz, Acetone-*d_6_*) *δ* 9.24 (s, 0.48H), 8.48 (bs, 0.49H), 8.23 (s, 1H), 7.74–7.71 (m, 4H), 7.48–7.42 (m, 6H), 6.87 (d, 1H, *J* = 8.0 Hz), 5.52 (d, 1H, *J* = 8.0 Hz), 4.77 (dd, 1H, *J* = 5.6, 1.3 Hz), 4.69–4.63 (m, 1H), 4.55 (t, 1H, *J* = 6.1 Hz), 3,82 (d, 2H, *J* = 5.9 Hz), 2.34–2.27 (m, 1H), 2.22–2.15 (m, 1H), 2.03–1.91 (m, 1H), 1.72–1.69 (m, 2H), 1.64–1.51 (m, 6H), 1.37–1.34 (m, 2H)., 1.08 (bs, 9H). ^13^C NMR (101 MHz, Acetone-*d_6_*) *δ* 160.8, 151.3.2, 146.5, 144.9, 137.3, 135.3, 134.5, 131.5, 129.5, 114.9, 99.8, 83.8, 82.4, 66.6, 63.9, 47.9, 39.3, 36.4, 33.9, 28.1, 26.7, 25.6, 25.2, 20.8. HRMS-ESI (*m*/*z*) [M+H]^+^ calcd. for C_32_H_42_N_3_O_5_Si: 576.2815, found: 576.2891.

**1-((1*R*,2*S*,3*R*,4*R*)-2,3-Dihydroxy-4-(hydroxymethyl)cyclopentyl)-4-(hydroxyamino)pyramid-in-2(1H)-one hydrochloride (9):**

To a solution of **22** (100 mg, 0.174 mmol) in MeOH (3.7 mL), HCl (1 M, 4.35 mL, 4.35 mmol) was added at 0 °C. The resulting reaction mixture was stirred for 4 h before evaporation of the volatiles. The residue was dissolved in water (15 mL) and washed with EtOAc (3 × 5 mL). The aqueous layer was concentrated under reduced pressure and then was co-evaporated 5 times with EtOH. The final residue was lyophilized to obtain pure compound **9** (42.7 mg, 82%). ^1^H NMR (400 MHz, D_2_O) δ 7.58 (d, 1H, *J* = 8.0 Hz), 5.96 (d, 1H, *J* = 8.0 Hz), 4.62 (q, 1H, *J* = 10.3 Hz), 4.16–4.12 (m, 1H), 3.87 (t, 1H, *J* = 4.4 Hz), 3.61–3.49 (m, 2H), 2.20–1.99 (m, 2H), 1.40 (q, 1H, *J* = 8.8 Hz). ^13^C NMR (101 MHz, D_2_O) δ 166.3, 153.4, 143.0, 92.1, 73.5, 71.4, 62.8, 62.1, 44.2, 26.7. HRMS-ESI (*m*/*z*) [M+H]^+^ calcd. for C_10_H_16_N_3_O_5_: 258.1045, found: 258.1084.

**4-(Hydroxyamino)-2′,3′-dideoxyuridine (12):**

To a solution of **23** (164 mg, 0.645 mmol) in pyridine (5 mL), 4-chlorophenyl phosphorodichloridate (0.157 mL, 0.967 mmol) and 1,2,4-triazole (133 mg, 1.93 mmol) were added dropwise at 0 °C. The mixture was stirred at room temperature for 5 days and then concentrated under reduced pressure. The resulting residue was dissolved in DCM (10 mL) and washed with H_2_O (2 × 10 mL) and with a 50% NaHCO_3_ solution (5 mL). The organic layer was clarified with Norit, dried over MgSO_4_, and filtered. The filtrate was evaporated to dryness in vacuo to yield **24** as a glassy residue. The crude compound **24** was dissolved in acetonitrile (13 mL) and NH_2_OH in H_2_O (50%, 0.25 mL) was added to the solution. The mixture was stirred for 2 h at room temperature to obtain crude compound **25** which was finally stirred overnight in a saturated solution of methanolic ammonia (10 mL) at room temperature. The volatiles were then evaporated under vacuum and the residue was purified by flash column chromatography (methanol/dichloromethane, 0/100 to 5/95) to obtain compound **12** (22 mg, 15 %). ^1^H NMR (400 MHz, MeOH) *δ* 8.30 (s, 1H, NH), 7.18 (d, 1H, *J* = 7.93 Hz), 6.06–6.03 (m, 1H), 5.58 (d, 1H, *J* = 7.93 Hz), 4.09–4.03 (m, 3H), 3.79–3.61 (m, 2H), 2.32–2.24 (m, 1H), 2.15–1.86 (m, 3H); ^13^C NMR (101 MHz, MeOD) *δ* 151.5, 145.6, 132.0, 98.9, 86.4, 82.2, 64.4, 32.2, 26.7. -ESI (*m*/*z*) [M+H]^+^ calcd. for C_9_H_14_N_3_O_4_ 228.0906, found 228.0980.

## 4. Conclusions

A series of thirteen 4-NHOH pyrimidine nucleoside analogs of NHC/molnupiravir (compounds **1**–**13**) were synthesized and evaluated in vitro for anti-SARS-CoV-2 activity in Vero cells. Unfortunately, none of them displayed significant activity up to 10 μM. These results emphasize, once more, the difficulty in designing antiviral nucleoside analogs for SARS-CoV-2, as a simple modification of a highly active compound can lead to the complete loss of antiviral potency.

## Figures and Tables

**Table 1:** Anti-SARS-CoV-2 activity evaluation of compounds **1**–**13** in Vero cells at 10 µM.

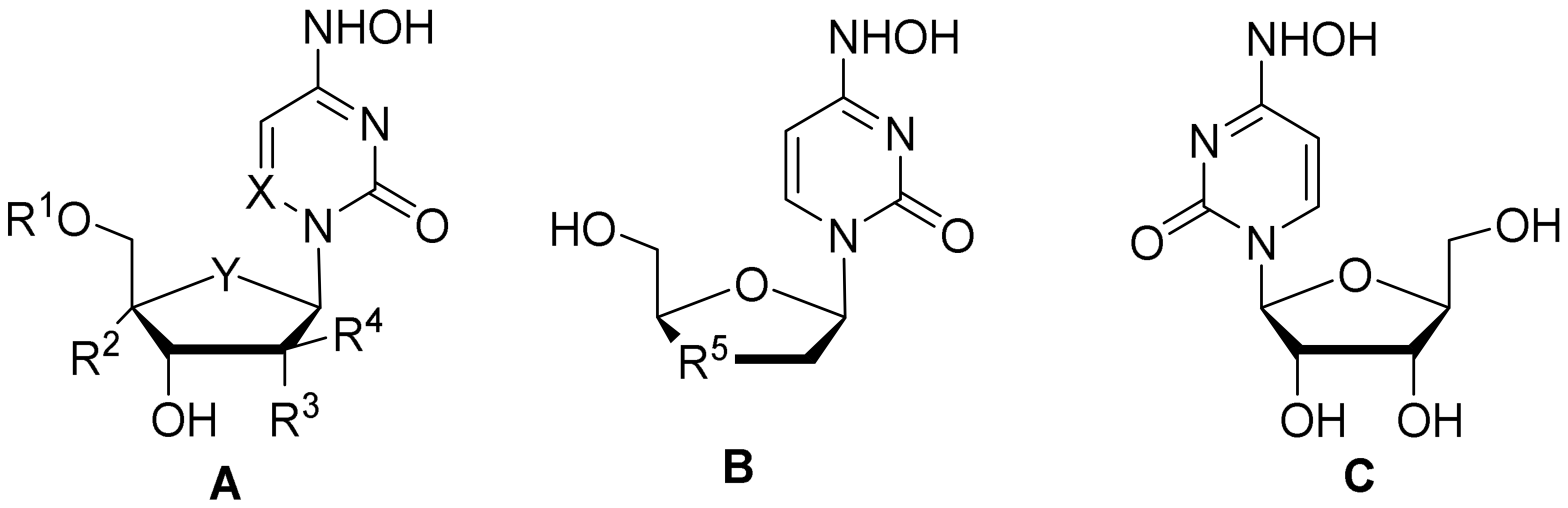									
Compound	Scaffold	R^1^	R^2^	R^3^	R^4^	R^5^	X	Y	% Viral Inhibition at 10 µM
**1**	**A**	H	H	H	H	*NA*	CH	O	13
**2**	**A**	H	H	F	F	*NA*	CH	O	0
**3**	**A**	H	H	F	H	*NA*	CH	O	34
**4**	**A**	H	H	OH	Me	*NA*	CH	O	0
**5**	**A**	H	H	F	Me	*NA*	CH	O	0
**6**	**A**	H	H	Cl	Me	*NA*	CH	O	9
**7**	**A**	Ac	F	OH	H	*NA*	CH	O	0
**8**	**A**	H	H	OH	H	*NA*	CH	Se	16
**9**	**A**	H	H	OH	H	*NA*	CH	CH_2_	28
**10**	**A**	H	H	OH	H	*NA*	N	O	33
**11**	**B**	*NA*	*NA*	*NA*	*NA*	O	*NA*	*NA*	0
**12**	**B**	*NA*	*NA*	*NA*	*NA*	CH_2_	*NA*	*NA*	22
**13**	**C**	*NA*	*NA*	*NA*	*NA*	*NA*	*NA*	*NA*	18
**NHC**	**A**	H	H	OH	H	*NA*	CH	O	99

*NA*: Not applicable.

## Data Availability

Data are contained within the article.
